# Guided endodontic treatment of multiple teeth with dentin dysplasia: a case report

**DOI:** 10.1186/s13005-020-00240-4

**Published:** 2020-11-17

**Authors:** Ralf Krug, Julian Volland, Sebastian Reich, Sebastian Soliman, Thomas Connert, Gabriel Krastl

**Affiliations:** 1 Department of Conservative Dentistry and Periodontology and Center of Dental Traumatology, University Hospital of Würzburg, Würzburg, Pleicherwall 2, 97070 Würzburg, Germany; 2 Department of Periodontology, Endodontology and Cariology, University Centre for Dental Medicine, University of Basel, Mattenstraße 40, 4058 Basel, Switzerland

**Keywords:** Guided endodontics, Pulp canal calcification, Dentin dysplasia, Root canal treatment, Template

## Abstract

**Background:**

To report the outcome of guided endodontic treatment (GET) of a case of dentin dysplasia with pulp canal calcification (PCC) and apical periodontitis based on the use of a 3D-printed template designed by merging cone-beam computed tomography (CBCT) and surface scan data.

**Case presentation:**

A 12-year old female with radicular dentin dysplasia type I (DD-1) presented for endodontic treatment. Radiography revealed PCC in all teeth and apical radiolucency in seven teeth (12, 15, 26, 31, 32, 36 and 46). Tooth 36 had the most acute symptoms and was thus treated first by conventional access cavity preparation and root canal detection. Despite meticulous technique, the distal and mesiolingual canals were perforated. The perforations were immediately repaired with mineral trioxide aggregate, and the decision was made to switch to guided endodontic treatment for the remaining 6 teeth. CBCT and intraoral surface scans were acquired and matched using coDiagnostix planning software (Dental Wings Inc.), the respective drill positions for root canal location were determined, and templates were virtually designed and 3D-printed. The template was positioned on the respective tooth, and a customized drill was used to penetrate the calcified part of the root canal and perform minimally invasive access cavity preparation up to the apical region. All root canals were rapidly and successfully located with the templates. At 1-year follow-up, clear signs of apical healing were present in all treated teeth.

**Conclusions:**

In patients with dentin dysplasia, conventional endodontic therapy is challenging. GET considerably facilitates the root canal treatment of teeth affected by dentin dysplasia.

## Background

Endodontic treatment of calcified pulp systems is challenging. Pulp canal calcification (PCC) is reported to occur after various luxation injuries at rates of 15–40% [1, 2]. Chronic irritation (e.g. caries), cervical pulpotomy, or restorative therapies are known to promote the apposition of hard tissues within the root canal [3–5]. It may also occur after orthodontic treatment [6] or in elderly patients with a high rate of physiological apposition of dentin [7]. Specifically, dentin dysplasia (DD), a kind of dentin malformation, also causes accelerated dentinal apposition [8, 9].

DD, a rare disturbance of dentin formation (incidence: 1:100,000), is an autosomal dominant hereditary disease caused by a coding malfunction of the dentin sialophosphoprotein gene. The disorder is characterized by apparently normal enamel but atypical dentin formation and abnormal pulp morphology [10]. Two types of DD are distinguished based on clinical, radiological and histological findings: type I (“radicular”) and type II (“coronal”), hereinafter referred to as DD-1 and DD-2 [11]. In both DD-1 and DD-2, the crowns of primary and permanent teeth mostly have a clinically and morphologically normal appearance and colour (in DD-2, however, the primary teeth are bluish amber-coloured). The affected teeth may exhibit abnormal mobility, premature exfoliation and, particularly in the case of DD-1, undeveloped or absent roots [12].

Radiographically, the pulp spaces may be narrowed and thus reduced in size or completely calcified. In DD-2, teeth in both dentitions often have thin roots but normal root lengths. Common radiologic signs include early pulp obliteration and thistle-tube shaped pulp chambers with multiple pulp stones in the absence of periapical radiolucencies [13]. Besides aberrant dentin deposition in the pulp chamber in both types (DD-1 and DD-2), most typically in DD-1 there is an increased incidence of periapical radiolucencies due to the infection of the root canal system [9, 13, 14]. To date, it is presumed that these teeth are highly susceptible to bacterial invasion due to various factors, including the presence of atypical dentin formation within a highly irregular pulp chamber, the lack of dentinal fluid, which induces enamel brittleness and, occasionally, micro cracks as a potential pathway for microorganisms, and aberrant dentin formation, which might affect the formation and structure of the enamel as well [15]. Alterations to the enamel, including micro cracks and increased dentin permeability in the case of DD-1, may facilitate bacterial invasion and lead to pulp necrosis and apical periodontitis. Such a crack was detected in a three-dimensional (3D) micro-computed tomographic study of a molar of a DD-1 patient, but the ability of this technology to detect fine cracks seems to be limited [15].

There is no available evidence on the occurrence of apical periodontitis in teeth with DD, but several case reports suggest that these teeth may be at risk of developing apical pathologies [10, 14–18].

**Fig. 1:**
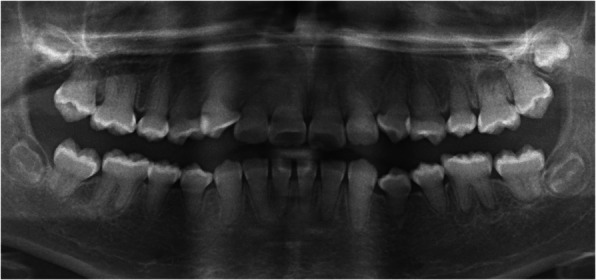
Panoramic view of the dentition of a 12-year old female with DD-1: Note the obliterated pulp chambers and root canals of reduced size in all teeth, and periapical radiolucencies in teeth 15, 12, 26, 36, 32, 31, 46

In the present case report, DD-1 was identified based on its specific radiologic features, including the characteristic root morphology, obliteration of the pulp chambers and root canals, and the presence of several periapical lesions in sound teeth (Figs. 1, 3). Typically, the roots appear shortened, blunted, and partially malformed [14, 16, 19, 20].

Endodontic treatment of calcified pulp systems (due to dental trauma or DD) is associated with a high risk of complications, such as root perforation, extensive dentinal hard tissue loss, and missing the root canal [14, 17]. Thus, straight-line access close to or through the incisal edge was emphasized as being a key to preventing technical endodontic failures in anterior teeth with PCC [21]. Guided endodontic treatment (GET) was recently introduced as a novel method of performing operator-independent guided straight-line access [22, 23]. Here, 3D data collected by cone-beam computed tomography (CBCT) were matched with surface scan data and used to fabricate a 3D drill guide. Once the drill paths were virtually planned, templates could be fabricated to safely locate the root canals. Other case reports and one case series have also shown the feasibility and reliability of GET in cases with apical periodontitis as a late complication of dental trauma [23–31]. To our knowledge, there is currently only one guided endodontics case report, which describes a promising 18-month outcome of a dens invaginatus malformation case treated, followed by endodontic surgery and root-end filling with mineral trioxide aggregate [30].

In DD cases in need of endodontic therapy, conventional orthograde endodontic treatment was reported to be potentially substitutable with periapical curettage and retrofilling, especially if the roots display normal length and development [32, 33]. In the case of DD-1, extraction of teeth with periapical radiolucencies might be favoured over endodontic therapy due to the above-mentioned technical challenges and anatomic limitations [34–36].

To date, only a few case reports of endodontic treatment attempts in patients with DD-1 have been published, and they have partially sobering outcomes [14, 37–39]. This is the first case report of a successful outcome of root canal treatment of multiple teeth in a patient with DD-1. GET prevented technical complications and enabled tooth retention in our patient.

## Case presentation

**Fig. 2:**
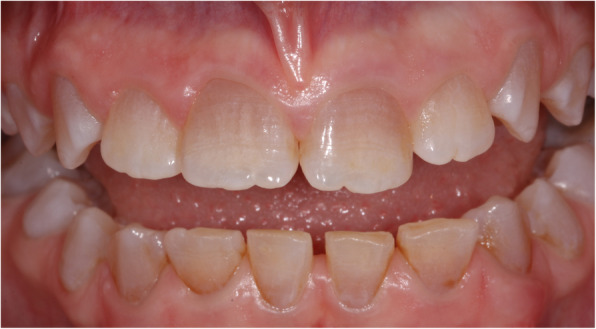
Clinically, the enamel appears normal but there is atypical dentin formation with abnormal pulp morphology

**Fig. 3:**
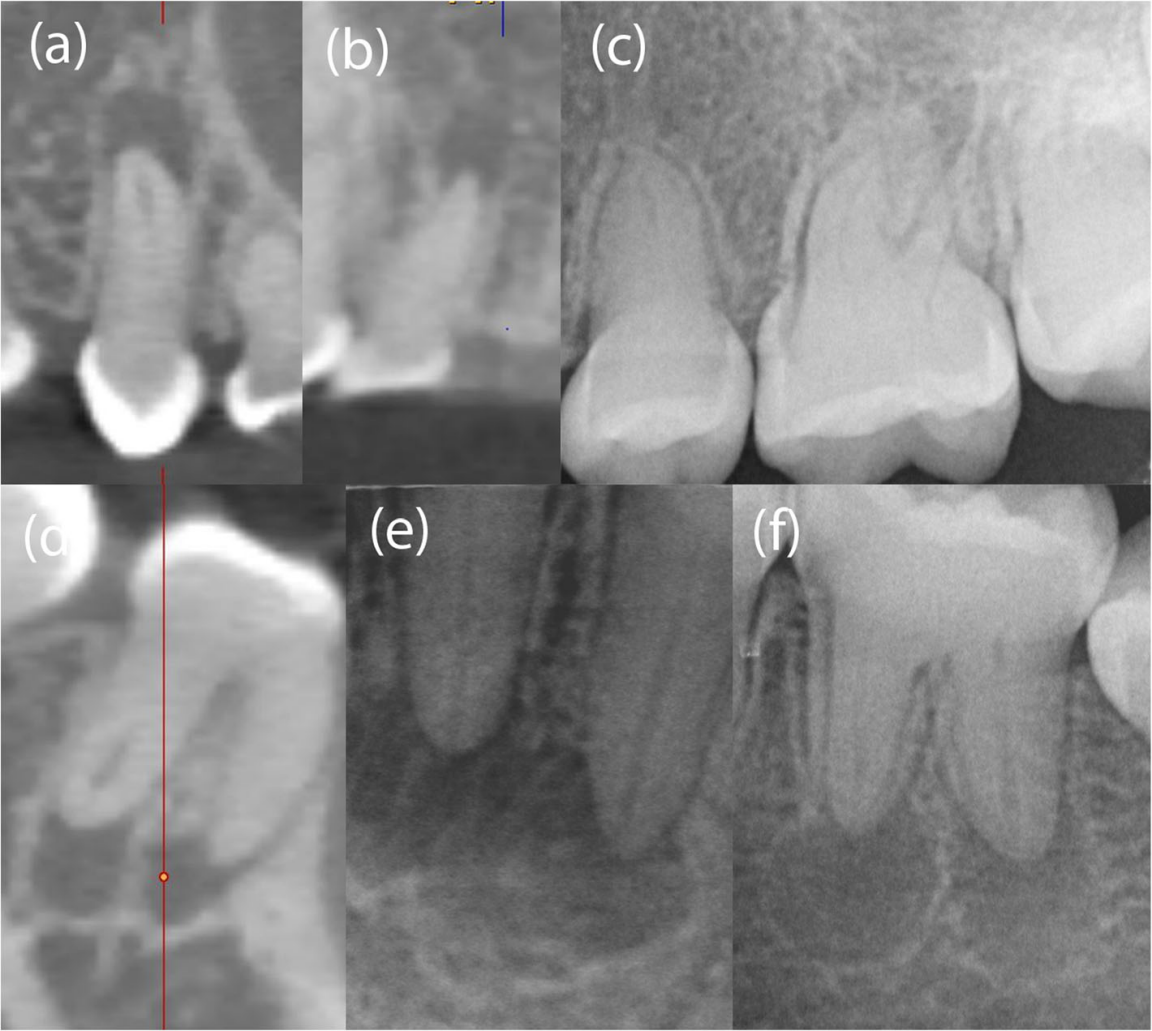
Periapical radiolucencies were detected in teeth 15, 12, 46 by CBCT imaging (**a**, **b**, **d**) and in teeth 26, 36, 32, 31 by periapical radiography (**c**, **e**, **f**)

A 12-year old female with DD-1 was referred to our dental clinic (Department of Conservative Dentistry and Periodontology and Center of Dental Traumatology, University Hospital of Würzburg, Germany) for endodontic treatment of multiple teeth. Due to the typical clinical and radiologic appearance of the teeth, no further attempts were made to confirm the clinical diagnosis of DD-1. The patient was not on any medication. Written informed consent for publication of their clinical details and clinical images was obtained from the parents of the patient. Clinically, the patient exhibited acute pain on percussion of tooth 36 and was diagnosed with symptomatic apical periodontitis. All of her teeth were unrestored and free of carious or non-carious loss of dental hard tissue. They showed regular enamel (Fig. 2), but aberrant dentin formation and irregular pulp morphology. Neither severe tooth mobility nor atypical tooth positions were observed. Periapical radiographs revealed PCC in all teeth and apical radiolucencies in seven teeth: 15, 12, 26, 36, 32, 31 and 46 (Fig. 3). The pulp spaces were reduced in size or completely calcified. Conventional endodontic treatment of tooth 36 was initiated due to acute symptoms. While attempting to locate the root canals, perforation of the distal and mesiolingual root canals occurred despite meticulous treatment under a microscope. The perforations were immediately repaired with mineral trioxide aggregate (ProRoot® MTA, Dentsply Sirona). Conventional root canal treatment was performed in two sessions after successful root canal location in this tooth.

**Fig. 4:**
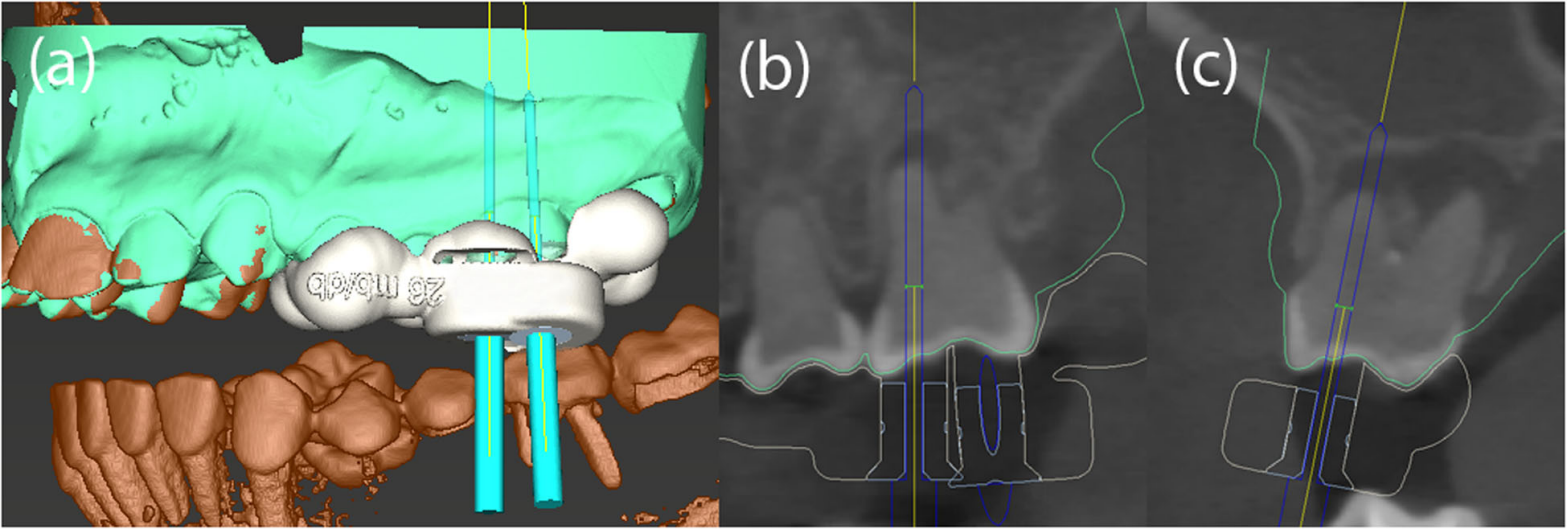
The drill paths for tooth 26 (e.g. mesio- and disto-buccal) were virtually planned using coDiagnostix software (Dental Wings Inc.) (**a**-**c**)

**Fig. 5:**
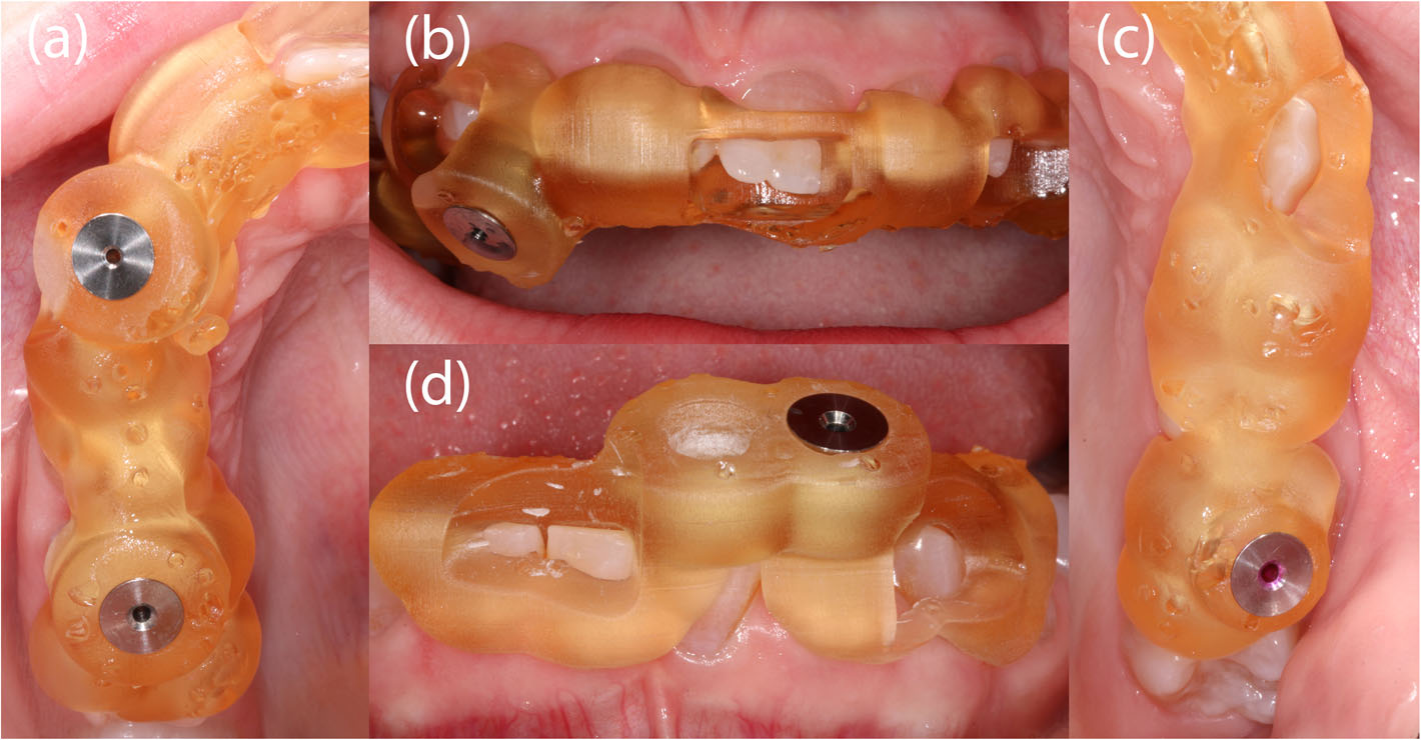
Intraoral photograph showing the inserted templates and drill sleeves (steco-system-technik GmbH) for teeth 15 and 12 (**a**, **b**), for the palatal root canal of tooth 26 (**c**), and for teeth 32 and 31 (**d**)

**Fig. 6:**
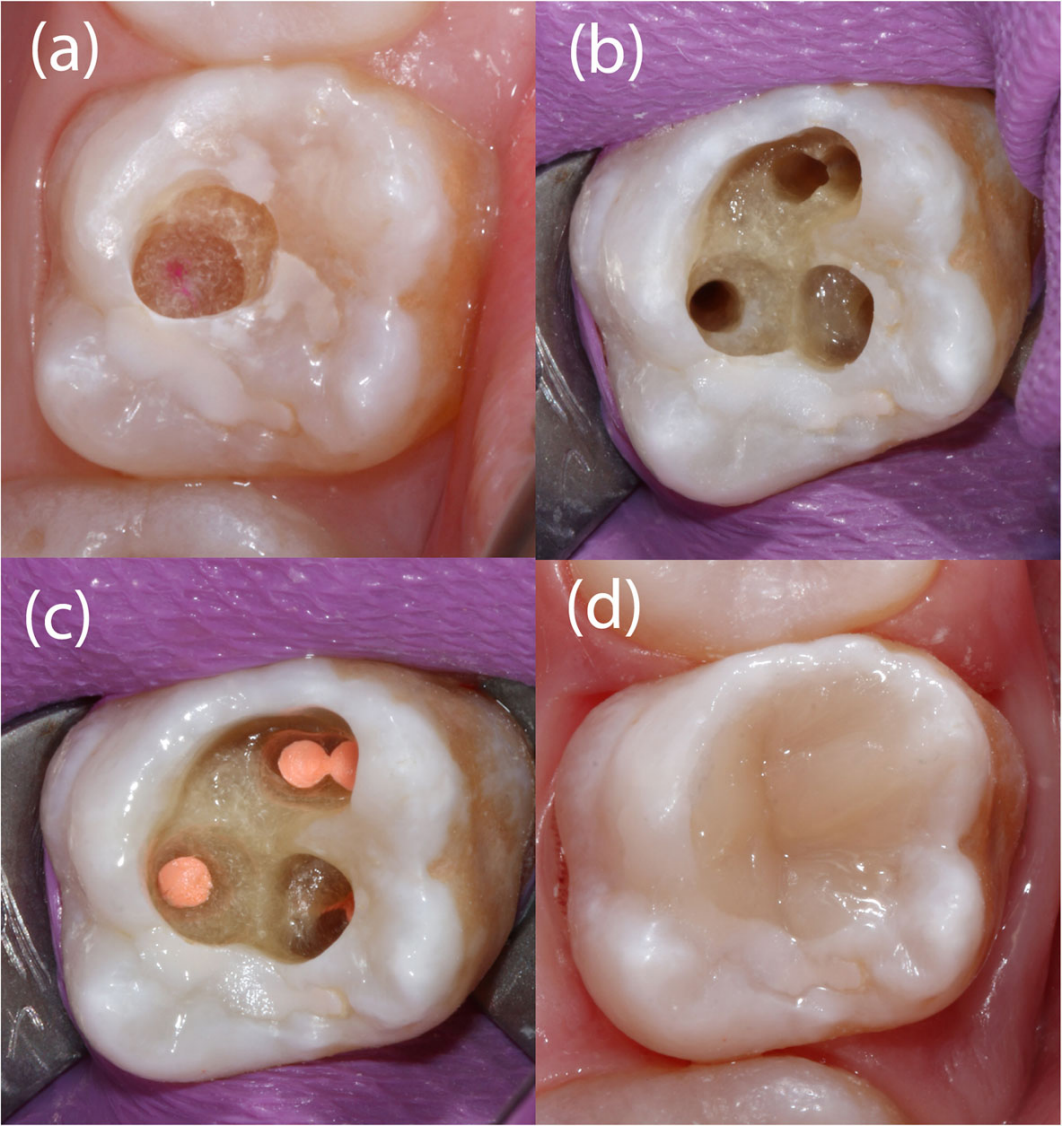
The enamel has to be removed step-by-step, targeting each root canal. Staining of the tip of the bur with the template in place on the tooth crown makes it possible to locate the entry point of the bur within the dentin (**a**). Photo taken during endodontic treatment of tooth 26, with palatal, disto-buccal and two mesio-buccal root canals (**b**). Subsequently, obturation was carried out using gutta-percha and sealer (AHplus, Dentsply Sirona) (**c**), and the access cavity was filled with composite (**d**)

**Fig. 7:**
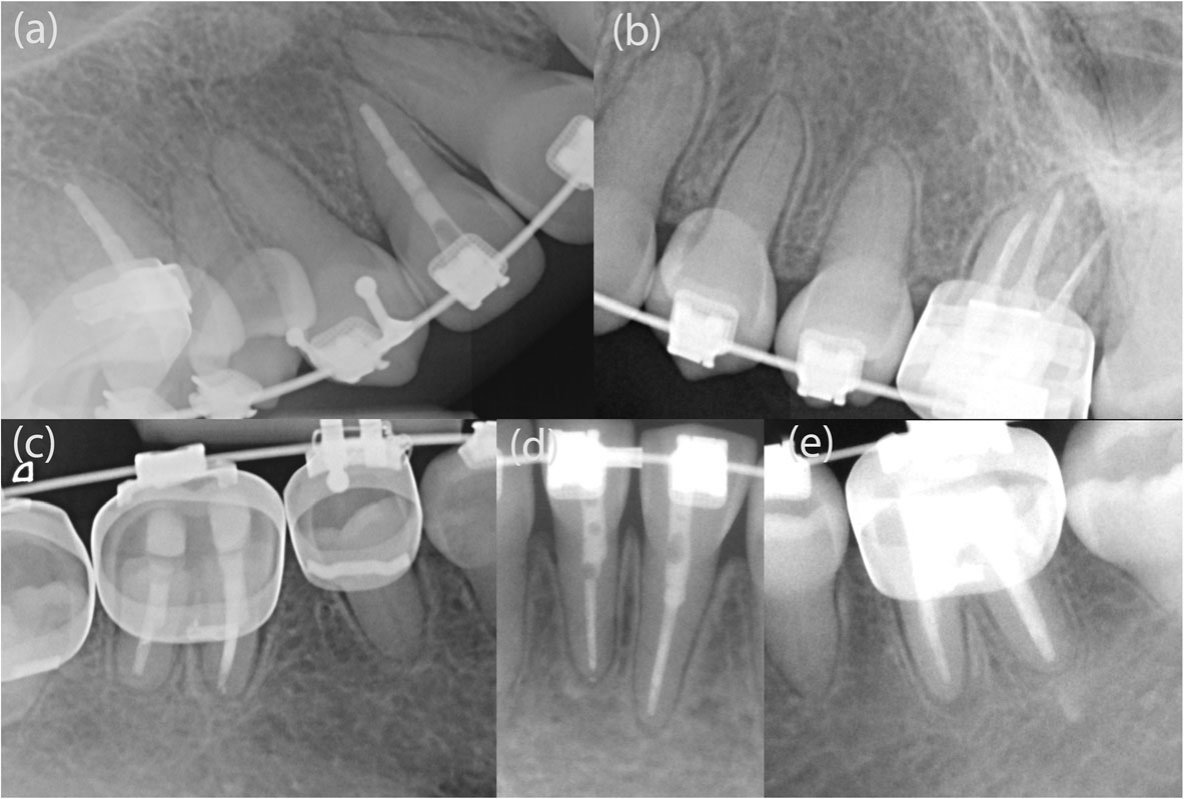
This 1-year follow-up radiograph showed size reduction of the apical lesions in endodontically treated teeth 12 (**a**), 36 (**e**), and 32 (**d**), and complete regression in 15 (**a**), 26 (**b**), 31 (**d**), and 46 (**c**)

Given the difficulty in locating the root canals, the operator switched to GET for the remaining 6 asymptomatic teeth. CBCT (3D Accuitomo 170, J. Morita Mfg. Corp.) and intra-oral surface scans (Cerec Omnicam, Dentsply Sirona GmbH) were acquired and matched using coDiagnostix planning software (Dental Wings Inc.). After the drill position for root canal location was determined (Fig. 4), a virtual template was designed. The corresponding surface-tessellation-language (STL) files were exported to a 3D printer (Form 2 Formlabs, Material: Dental SG Resin, Formlabs Inc., Somerville, MA, USA) for template fabrication. After inserting the drill sleeves (steco-system-technik GmbH), the template was positioned on the respective teeth requiring endodontic treatment (Fig. 5). A customized drill (diameter = 1.0 mm, steco-system-technik GmbH) was used to create a minimally invasive access to the calcified root canal in the apical third of the root. The orifices of all root canals were rapidly and successfully located. Endodontic therapy consisted of mechanical preparation using nickel-titanium rotary files (Mtwo®, VDW GmbH), sonic irrigant activation was performed using Eddy® tips (VDW GmbH) and sodium hypochlorite (3%), followed by warm vertical gutta-percha obturation with an epoxy resin sealer (AH Plus®, Dentsply Sirona). Finally, the access cavities were restored with composite resin (Fig. 6). At the 1-year follow-up examination, the root-filled teeth were symptom-free. Radiography revealed signs of apical lesion size reduction in teeth 36, 32, and 12. Further follow-ups are needed to clarify the ultimate endodontic outcome. Complete healing of apical periodontitis was obtained in teeth 15, 26, 31, and 46 (Fig. 7). Improvement of tooth mobility was observed in all endodontically treated teeth compared with the initial mobility.

## Discussion

This is the first case report of the use of GET to prepare minimally invasive access cavities for root canal location in a patient with DD-1. All of the patient’s teeth showed radiographic signs of PCC, and apical periodontitis was detected in a total of seven teeth. Initially, endodontic therapy was performed on tooth 36 using the conventional approach, resulting in two perforations, whereas GET on the remaining affected teeth led to a successful outcome. The 1-year follow-up examination revealed signs of healing of the lesions of endodontic origin in all root canal treated teeth.

Patients with DD-1 frequently have clinical signs of apical periodontitis, spontaneous abscess formation or tooth hypermobility related to an unfavourable crown-to-root ratio. These conditions pose diagnostic challenges, and the recommended treatment possibilities are unspecific and limited [40]. For management of such affected teeth with short roots, hypermobility, PCC, and periapical lesions, it was recommended to focus on two extreme options: either monitor without any treatment or extract the tooth as the last resort [13, 18]. Endodontic instrumentation and obturation, an option in teeth without extremely short roots [13], is often believed to be unfeasible because of abnormal pulp canal morphology. Ramifications, the occurrence of pulp stones and various types of hard tissue formations within the root canal system are typical characteristics [18]. In a questionnaire-based evaluation of one DD-1 case history, 90.6% of a total of 64 dentists with different specialities and experience levels chose to monitor multiple periapical radiolucencies in teeth with pulp necrosis instead of performing root canal treatment (3.1%) or extraction (6.2%) [41]. Likewise, in a case report of a 7-year-old female DD-1 patient with a 6-year follow-up, the authors preferred extracting permanent teeth due to extensive mobility. Extraction was suggested for teeth with pulp necrosis and periapical abscess, and endodontic treatment was considered to be contraindicated in those with severe calcification of the pulp [35]. Furthermore, endodontic therapy might be limited in young children, as an early age of the patient is associated with poor compliance in such difficult and lengthy treatment.

Root canal location in calcified teeth with DD might be even more challenging than in those with calcification after trauma or caries. In teeth with DD-1, calcification of the pulp space seems to follow a different yet unknown pathophysiological mechanism. The process of calcification occurs soon after or even prior to tooth eruption [42], and it does not necessarily start in the coronal part of the tooth in association with subsequent gradual narrowing of the root canals, as is often the case after luxation injuries. Instead, teeth with DD-1 seem to have variable expressivity, ranging from total obliteration of the endodontic space to calcifications in specific regions in the apical or coronal part of the tooth [42]. Thus, the presence of a narrow root canal in the apical part of the root, as reported for teeth with pulp canal calcifications [43], might not apply for teeth with DD-1.

Another aspect which makes root canal location more difficult is dentin morphology. In patients with DD-1, the teeth have softer dentin that lacks a regular tubular morphology and may contain irregular channels [44]. Thus, regardless of the location of the root canal, files with cutting tips, which are frequently used to scout root canals and penetrate calcifications, might also penetrate the soft dentin and increase the risk of perforation.

After the occurrence of two perforations during conventional root canal treatment to manage a mandibular molar with symptoms in the present case, it was considered better to switch to GET for safe endodontic management of the remaining teeth. The root canals in all teeth could be successfully located, prepared and adequately root-filled using GET approach. Nevertheless, the favourable results after 1 year, as reflected by clear signs of periapical healing, do not guarantee long-term success. While the success rates of primary root canal treatment in teeth with preoperative apical periodontitis generally may reach 74% [45], little is known about the fate of root-canal-treated teeth in patients with DD-1. Three case reports describe successful conventional endodontic management, partially completed with apical surgery [14, 37, 38]. Successful endodontic treatment with complete healing of periapical lesions in 16 teeth was solely observed in a 22-year old female with DD-1, who was monitored for up to 3 years [14].

Most published cases had rather sobering outcomes after different therapy approaches. This includes a switch to apical surgery after an unsuccessful endodontic treatment attempt [39] or leaving teeth with periapical lesions untreated [18, 35] or tooth extraction, particularly in teeth with short roots [10, 19, 20, 34, 36, 46]. The prognosis of endodontically treated teeth with DD might be described as uncertain. It may be hypothesized that because the increased dentin permeability allows persistent bacterial penetration, the chances of complete healing of periapical lesions may be limited, even in adequately root-filled teeth. Further, reversal of healing might be more likely than in teeth without DD-1. In the present case reported here, a promising outcome of endodontic treatment was observed at 1-year follow-up. However, further follow-ups are needed to monitor the healing process and to identify new lesions of endodontic origin which may occur in other teeth. In teeth with DD-1 there might be an increased risk for reinfection of the root canal space after endodontic treatment due to the softer and more permeable dentin and the presence of micro cracks [15]. Thus, all attempts should be made to optimally seal the root canal below the marginal bone with adhesive materials.

From a technical perspective, the GET approach has proven its worth in the present case, where multiple teeth were endodontically treated. GET is generally considered to be clinically useful for facilitating the root canal treatment of anterior and posterior teeth with PCC and apical periodontitis. Various ex-vivo study results revealed that GET is a highly precise and time-saving technique of root canal location [47–49]. However, in severely curved root canals, GET may fail to negotiate the apical canal curvature. For such cases, apicectomy may be a viable alternative treatment approach. Interestingly, a guided microsurgical technique was introduced to allow predefined osteotomy and root resection [50].

Prospectively, advanced guided endodontics based on a new computer-aided dynamic navigation technology may soon be available for cases where multiple root canal treatments have to be performed in calcified teeth. However, further investigations are needed to confirm the promising preliminary results of this novel technology [51, 52].

## Conclusions

Conventional endodontic treatment of teeth affected by dentin dysplasia is challenging but can be facilitated by GET. As demonstrated in multiple teeth of the DD-1 patient described in this case report, GET is a safe and clinically feasible technique that prevents root perforation and enables tooth retention.

## Data Availability

The datasets used and/or analysed during the current case report are available from the corresponding author on reasonable request.

## Abbreviations

3D: Three-dimensional
AP: Apical periodontits
CBCT: Cone-beam computed tomography
DD: Dentin dysplasia
GET: Guided endodontic treatment
PCC: Pulp canal calcification
STL: Surface-tessellation-language
